# Severe Fatigue in Long COVID: Web-Based Quantitative Follow-up Study in Members of Online Long COVID Support Groups

**DOI:** 10.2196/30274

**Published:** 2021-09-21

**Authors:** Maarten Van Herck, Yvonne M J Goërtz, Sarah Houben-Wilke, Felipe V C Machado, Roy Meys, Jeannet M Delbressine, Anouk W Vaes, Chris Burtin, Rein Posthuma, Frits M E Franssen, Bita Hajian, Herman Vijlbrief, Yvonne Spies, Alex J van 't Hul, Daisy J A Janssen, Martijn A Spruit

**Affiliations:** 1 REVAL Rehabilitation Research Center BIOMED Research Institute Faculty of Rehabilitation Sciences, Hasselt University Diepenbeek Belgium; 2 Department of Research and Development Ciro Horn Netherlands; 3 Nutrim School of Nutrition and Translational Research in Metabolism Faculty of Health, Medicine and Life Sciences Maastricht University Maastricht Netherlands; 4 Department of Respiratory Medicine Maastricht University Medical Centre Maastricht Netherlands; 5 Lung Foundation Netherlands Amersfoort Netherlands; 6 Department of Pulmonary Disease Radboud University Medical Center Nijmegen Netherlands; 7 Department of Health Services Research, Care and Public Health Research Institute Faculty of Health, Medicine and Life Sciences Maastricht University Maastricht Netherlands

**Keywords:** COVID-19, SARS-CoV-2, long COVID, post-COVID-19 syndrome, post-acute sequelae of COVID-19, fatigue, post-viral fatigue, pandemic, online health, mental health, online support

## Abstract

**Background:**

Fatigue is the most commonly reported symptom in patients with persistent complaints following COVID-19 (ie, long COVID). Longitudinal studies examining the intensity of fatigue and differentiating between physical and mental fatigue are lacking.

**Objective:**

The objectives of this study were to (1) assess the severity of fatigue over time in members of online long COVID peer support groups, and (2) assess whether members of these groups experienced mental fatigue, physical fatigue, or both.

**Methods:**

A 2-wave web-based follow-up study was conducted in members of online long COVID peer support groups with a confirmed diagnosis approximately 3 and 6 months after the onset of infectious symptoms. Demographics, COVID-19 diagnosis, received health care (from medical professionals or allied health care professionals), fatigue (Checklist Individual Strength–subscale subjective fatigue [CIS-Fatigue]; 8-56 points), and physical and mental fatigue (self-constructed questions; 3-21 points) were assessed. Higher scores indicated more severe fatigue. A CIS-Fatigue score ≥36 points was used to qualify patients as having severe fatigue.

**Results:**

A total of 239 patients with polymerase chain reaction/computed tomography–confirmed COVID-19 completed the survey 10 weeks (SD 2) and 23 weeks (SD 2) after onset of infectious symptoms, respectively (T1 and T2). Of these 239 patients, 198 (82.8%) were women; 142 (59.4%) had no self-reported pre-existing comorbidities; 208 (87%) self-reported being in good health before contracting COVID-19; and 62 (25.9%) were hospitalized during acute infection. The median age was 50 years (IQR 39-56). The vast majority of patients had severe fatigue at T1 and T2 (n=204, 85.4%, and n=188, 78.7%, respectively). No significant differences were found in the prevalence of normal, mild, and severe fatigue between T1 and T2 (*P*=.12). The median CIS-Fatigue score was 48 points (IQR 42-53) at T1, and it decreased from T1 to T2 (median change: –2 points, IQR –7 to 3; *P*<.001). At T1, a median physical fatigue score of 19 points (IQR 16-20) and a median mental fatigue score of 15 points (IQR 10-17) were reported; these scores were lower at T2 for physical but not for mental fatigue (median change for physical fatigue –1 point, IQR –3 to 0, *P*<.001; median change for mental fatigue 0 points, IQR –3 to 3, *P*=.52). At the time of completing the follow-up survey, 194/239 (81.2%) and 164/239 (68.6%) of all patients had received care from at least one medical professional and one allied health care professional, respectively.

**Conclusions:**

Fatigue in members of online long COVID support groups with a confirmed COVID-19 diagnosis decreases from 10 to 23 weeks after onset of symptoms. Despite this, severe fatigue remains highly prevalent. Both physical and mental fatigue are present. It remains unclear whether and to what extent fatigue will resolve spontaneously in the longer term.

**Trial Registration:**

Netherlands Trial Register NTR8705; https://www.trialregister.nl/trial/8705.

## Introduction

As the current COVID-19 pandemic continues to evolve, its impact becomes apparent. Clinical studies of hospitalized, laboratory-confirmed patients have shown that the acute phase of COVID-19 is characterized by a large array of respiratory and non-respiratory symptoms [1]. Over time, it has become clear that not all previously hospitalized patients fully recover from these symptoms in the months after the infection [2,3]. In addition, nonhospitalized patients can present persistent complaints months after the onset of infection-related symptoms [4]. These long-lasting symptoms after COVID-19 are referred to as *long COVID* [5], “a condition whereby affected individuals do not recover for several weeks or months following the onset of symptoms suggestive of COVID-19” [6], and they have a major impact on patients’ quality of life (QoL) [7,8], care dependency [9], work participation [10,11], day-to-day activities, and physical functioning [12-14].

Fatigue, defined as “a subjective, unpleasant symptom which incorporates total body feelings ranging from tiredness to exhaustion creating an unrelenting overall condition which interferes with individuals’ ability to function to their normal capacity” [15], is the most commonly reported symptom in patients with long COVID [2-4,16]. Similarly, other infections, such as severe acute respiratory syndrome (SARS) [17,18], Middle East respiratory syndrome (MERS) [19], and Q fever [20] have previously been linked to long-term fatigue, often referred to as postviral fatigue syndrome. Existing literature suggests that fatigue has several clinical presentations. A common distinction is made between physical fatigue (ie, difficulty performing physical activities) and mental fatigue (ie, difficulties concentrating and performing cognitive tasks) [21].

To date, longitudinal studies that examine fatigue intensity in patients with long COVID are lacking. Moreover, it is not known whether patients experience mostly mental or physical fatigue during and after the infection. Therefore, the objectives of this study were to (1) assess the severity of fatigue over time in members of online long COVID peer support groups; and (2) assess whether members of online long COVID peer support groups experience mental fatigue, physical fatigue, or both. We hypothesized that fatigue would be common and persistent and that both physical and mental fatigue would be present in patients with long COVID.

## Methods

### Study Design and Participants

This study is a prospective web-based survey of members of two Facebook peer support groups for patients with long COVID in the Netherlands (approximately 11,000 members; [22]) and Flanders (Belgium, approximately 1200 members; [23]), and a panel of approximately 1200 people who registered at a website of the Netherlands Lung Foundation (*coronaplein* [24]), an online platform providing additional information, advice, and peer support. Note that these totals represent the number of members of each group at the period of data collection. Between June 4 and June 11, 2020 (the time point of completing the first survey [T1]), members were invited to complete a web-based survey. Participants who completed the first survey [4,8,9] and who agreed to be contacted for a follow-up study received a second survey between August 31 and September 8, 2020 (ie, approximately three months after the first survey; the time point of completing the second survey [T2]). Ethical approval for this study was waived by the medical ethics committee of Maastricht University because the Medical Research Involving Human Subjects Act (WMO) does not apply to this study (METC2020-1978 and METC2020-2254). The medical ethics committee of Hasselt University formally judged and approved the study (MEC2020/041). Digital informed consent was obtained twice from all respondents (at the start of each survey). Exclusion criteria were intensive care unit (ICU) admission during the acute phase of infection, an onset of symptoms before January 1, 2020, being in the acute phase of COVID-19 when answering the first survey (ie, onset of infectious symptoms less than 3 weeks before filling out the first survey [25]), or an incomplete survey. Cross-sectional and follow-up data from this study on persistent symptoms, QoL, care dependency, construct-validity of the post–COVID-19 functional status scale, and information and care needs of members of online long COVID peer support groups have been published before [4,8,9,11,26,27]. This 2-wave web-based follow-up study was registered at the Netherlands Trial Registry (NTR8705). The Strengthening the Reporting of Observational Studies in Epidemiology (STROBE) checklist was used to guide reporting [28]. Of note: the current study focusses on patients with a confirmed COVID-19 diagnosis (ie, test-diagnosed cases). The results for the patients without a confirmed COVID-19 diagnosis are presented in Multimedia Appendix 1.

### Assessment via Web-Based Surveys

The survey was developed in close collaboration with scientists, methodologists, health care professionals and COVID-19 patients from the long COVID peer support groups (the Netherlands and Flanders). It was digitalized by ASolutions [29] and was made available via their online platform. The survey consisted of general questions regarding demographics, clinical characteristics, and standardized questionnaires, including a fatigue questionnaire.

#### Demographic and Clinical Characteristics

Respondents received questions regarding demographical aspects such as gender, age, weight, height, educational level (low/medium/high; classification according to the International Standard Classification of Education 2011 [30]), and married/living with a partner (yes/no). In addition, the following clinical characteristics were assessed via self-report: pre-existing comorbidities (see Multimedia Appendix 2), health status (good/moderate/poor) during the infection and at the moment of completing the surveys, date of symptom onset, symptoms during acute phase of COVID-19 and at the moment of completing the surveys (see Multimedia Appendix 2), COVID-19–related hospitalization, and COVID-19 diagnosis (based on reverse transcription polymerase chain reaction (PCR) test or computed tomography (CT) scan of the thorax/symptom-based medical diagnosis by a physician/no formal test or diagnosis). Based upon the latter, patients were classified as either “test-diagnosed” COVID-19 (PCR or CT) or “presumed” COVID-19 (physician-diagnosed or no formal diagnosis/testing).

#### Received Health Care

Information regarding received health care (yes/no) by a medical professional (eg, medical specialist; general practitioner [GP]; nurse) or an allied health care professional (AHP; eg, physiotherapist [PT]; psychologist; occupational therapist [OT]; dietician; speech and language therapist) was recorded.

#### Standardized Fatigue Questionnaire

Fatigue, the primary outcome measure, was measured using a subscale of the Checklist Individual Strength (CIS). The Checklist Individual Strength–subscale subjective fatigue (CIS-Fatigue) is a standardized questionnaire [31,32] with high internal consistency and test-retest reliability; good discriminant, concurrent and criterion validity; and ability to detect change in subjective fatigue [33-37]. The questionnaire consists of 8 items scored on a 7-point Likert scale. Scores range from 8 to 56 points, and a higher score indicates more clinical symptoms of general fatigue (see Multimedia Appendix 3 for the CIS-Fatigue questionnaire) [31,32]. Based upon validated cutoff values, individuals can be classified as having normal (≤26 points), mild (27-35 points), and severe (≥36 points) fatigue [31-33].

#### Self-constructed Physical and Mental Fatigue Questions

A total of 3 self-constructed questions (all part of the CIS-Fatigue subscale) were used to evaluate physical fatigue (“*Physically* I feel exhausted,” “*Physically* I feel I am in a bad condition,” and “*Physically* I feel in a good shape”). In addition, to differentiate between physical and mental fatigue, 3 questions were constructed in which the word “physically” was replaced by the word “mentally” (“*Mentally* I feel exhausted,” “*Mentally* I feel I am in a bad condition,” and “*Mentally* I feel in a good shape,” respectively) to estimate mental fatigue. The physical and mental fatigue questions were scored on a 7-point Likert scale, with scores ranging from 3 to 21 points. A higher score indicates worse physical and mental fatigue, respectively. These self-constructed physical and mental fatigue questions and explanations of the scoring are reported in Multimedia Appendix 4.

### Statistical Analyses

Data are presented as means and standard deviations, medians, and interquartile ranges or as frequencies and proportions, where appropriate. Differences over time were analyzed by a paired *t*-test (or Wilcoxon signed-rank test) in continuous data and a McNemar test (or McNemar-Bowker test) in categorical data. If significant, a post hoc comparison of the McNemar-Bowker test was performed, and significant Bonferroni-adjusted *P* values were generated as corrections for multiple comparison. Statistical analyses were conducted using SPSS 25.0 (IBM Corporation). Figures were generated via GraphPad Prism 8.3.5 (GraphPad Software) and SankeyMATIC [38]. The level of significance was set at .01 for all statistical tests (two-tailed).

## Results

### Participants’ Inclusion

In total, 2159 members of online long COVID peer support groups filled out the first survey, of which 220 were excluded for being in the acute phase of COVID-19 (n=14), ICU admission during the acute phase of COVID-19 (n=15), onset of symptoms before January 1, 2020 (n=8), and an incomplete first survey (n=183). From the 1939 patients who were included, 1556 consented to be approached for follow-up research, of which 1005 (64.6%) completed the second survey. Patients who did not respond to the second survey were younger and more often had a presumed COVID-19 diagnosis. Further details can be found in a previously published paper [11]. The 1005 patients completed the surveys on average 11.3 weeks (SD 2.2) and 23.5 weeks (SD 2.2) after onset of symptoms (T1 and T2, respectively). Overall, 239 test-diagnosed (hospitalized, n=62, and nonhospitalized, n=177) and 766 presumed (physician-diagnosed, n=454, and patients with no formal diagnosis/testing, n=312) patients with COVID-19 participated in this 2-wave web-based survey (see Multimedia Appendix 5 for the flowchart).

### Demographical and Clinical Characteristics

Patients with confirmed COVID-19 were mostly middle-aged women (median age 50.0 years, IQR 39.0-56.0; 198/239 women, 82.8%) with a BMI indicating slight overweight (median BMI 26.0 kg/m^2^, IQR 23.4-30.5), and they completed the first (T1) and second (T2) survey on average 10.4 weeks (SD 2.4) and 22.6 weeks (SD 2.4) after onset of symptoms. Approximately 1 out of 4 patients (62/239, 25.9%) was hospitalized during the acute phase of COVID-19. The majority of respondents had no self-reported comorbidities (142/239, 59.4%) and good self-reported health status before the infection (208/239, 87%). Moreover, at T1 and T2, a minority of respondents self-reported good health (22/239, 9.2%, and 40/239, 16.7%, respectively [11]). Furthermore, patients retrospectively reported a median of 15 symptoms (IQR 11-18) during the acute phase of COVID-19, and 6 symptoms (IQR 4-9) and 6 symptoms (IQR 3-8) at T1 and T2, respectively. All details regarding patient characteristics can be found in Table 1.

**Table 1:** Characteristics of patients with confirmed COVID-19 (n=239).

Characteristic		Value
Women, n (%)		198 (82.8)
Age (years), median (IQR)		50.0 (39.0-56.0)
BMI (kg/m^2^), median (IQR)		26.0 (23.4-30.5)
Time between onset of symptoms and T1^a^ survey (weeks), mean (SD)		10.4 (2.4)
Time between onset of symptoms and T2^b^ survey (weeks), mean (SD)		22.6 (2.4)
Married/living with partner, n (%)		173 (72.4)
**Educational level, n (%)**		
	Low	6 (2.5)
	Medium	126 (52.7)
	High	107 (44.8)
**Pre-existing comorbidities, n (%)**		
	None	142 (59.4)
	1	62 (25.9)
	≥2	35 (14.6)
**Health status before infection, n (%)**		
	Good	208 (87)
	Moderate	28 (11.7)
	Poor	3 (1.3)
**Health status at T1, n (%)**		
	Good	22 (9.2)
	Moderate	156 (65.3)
	Poor	61 (25.5)
**Number of symptoms, median (IQR)**		
	During acute infection	15 (11-18)
	At T1	6 (4-9)
	At T2	6 (3-8)
Hospitalized during acute infection, n (%)		62 (25.9)

^a^T1: time of completing the first survey.

^b^T2: time of completing the second survey.

### Received Health Care

During the first 10 weeks after the onset of symptoms, 2 out of 3 patients (157/239, 65.7%) received or sought care from at least one medical professional (GP: 139/239, 58.2%; medical specialist: 73/239, 30.5%; nurse: 18/239, 7.5%), whereas 1 out of 3 (90/239, 37.7%) received or sought care from at least one allied health care professional (PT: 76/239, 31.8%; psychologist: 27/239, 11.3%; OT: 7/239, 2.9%, dietician: 25/239, 10.5%; and speech and language therapist: 6/239, 2.5%). The cumulative proportion of patients who received care from a medical professional and allied health care professional at T2 respectively increased significantly to 81.2% (194/239; GP: 170/239, 71.1%; medical specialist: 131/239, 54.8%; nurse: 32/239, 13.4%; all *P*<.001) and 68.6% (164/239; PT: 157/239, 65.7%; psychologist: 55/239, 23%; OT: 27/239, 11.3%; dietician, 51/239, 21.3%; speech and language therapist, 21/239, 8.8%; all *P*<.001). Furthermore, the cumulative proportion of patients who participated in an interdisciplinary rehabilitation program (in- or outpatient) increased significantly from T1 to T2 (10/239, 4.2%, to 32/239, 13.4%, respectively; *P*<.001). Details regarding received health care can be found in Table 2.

**Table 2:** Fatigue-related measures and received health care in patients with confirmed COVID-19 at on average 10 weeks (T1) and 23 weeks (T2) after onset of symptoms (n=239).

					Value					
				T1^a^		T2^b^		*P* value		
**Fatigue-related measures**										
	General fatigue (points on CIS-Fatigue^c^ scale), median (IQR)				48 (42-53)		46 (37-50)		<.001	
	Severe fatigue, n (%)				204 (85.4)		188 (78.7)		.03	
	Mental fatigue (points on self-constructed questions), median (IQR)				15 (10-17)		14 (10-17)		.52	
	Physical fatigue (points on self-constructed questions), median (IQR)				19 (16-20)		18 (14-19)		<.001	
**Received health care from a medical professional, n (%)**										
	**Received care from ≥1 medical professionals**				157 (65.7)		194 (81.2)		<.001	
		General practitioner		139 (58.2)		170 (71.1)		<.001		
		Medical specialist		73 (30.5)		131 (54.8)		<.001		
		Nurse		18 (7.5)		32 (13.4)		<.001		
**Received health care from an allied health care provider, n (%)**										
	**Received care from ≥1 allied health care providers**				90 (37.7)		164 (68.6)		<.001	
		Physiotherapist		76 (31.8)		157 (65.7)		<.001		
		Psychologist		27 (11.3)		55 (23)		<.001		
		Occupational therapist		7 (2.9)		27 (11.3)		<.001		
		Dietician		25 (10.5)		51 (21.3)		<.001		
		Speech and language therapist		6 (2.5)		21 (8.8)		<.001		
Rehabilitation (in- or outpatient), n (%)					10 (4.2)		32 (13.4)		<.001	

^a^T1: time of completing the first survey.

^b^T2: time of completing the second survey.

^c^CIS-Fatigue: Checklist Individual Strength–subscale subjective fatigue.

### Standardized Fatigue Questionnaire

**Figure 1:**
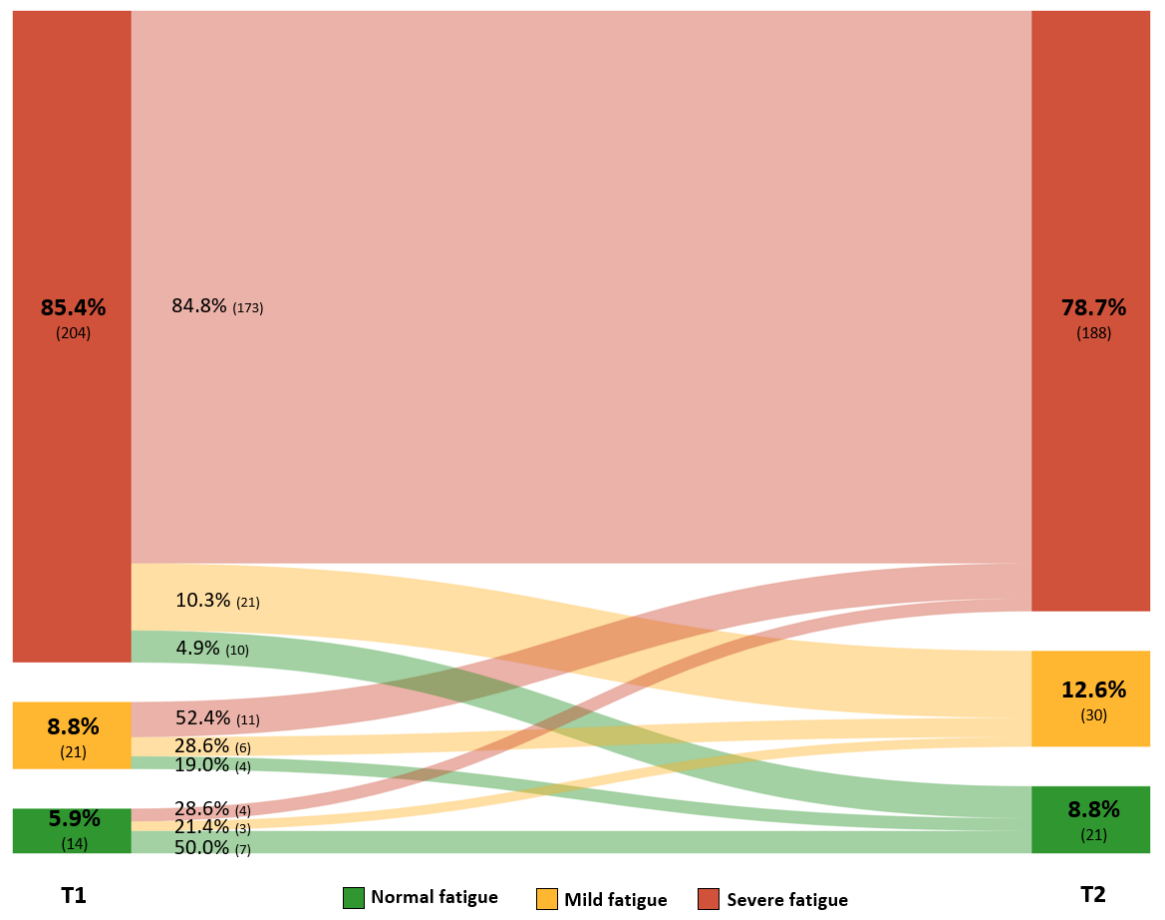
Prevalence and change in fatigue in patients with long COVID who have confirmed COVID-19, measured using the CIS-Fatigue scale at on average 10 (T1) and 23 (T2) weeks after onset of symptoms (n=239). The width of the lines is proportional to the flow rate. No significant change in the prevalence of normal (≤26 points), mild (27-35 points), or severe (≥36 points) fatigue was found between T1 and T2 (McNemar-Bowker test, *P*=.12). CIS-Fatigue: Checklist Individual Strength–subscale subjective fatigue; T1: time of completing the first survey; T2: time of completing the second survey.

Patients with confirmed COVID-19 reported a median CIS-Fatigue score of 48 points (IQR 42-53) at T1. The majority (204/239, 85.4%) reported severe fatigue at approximately 3 months after the onset of COVID-19 symptoms. The median CIS-Fatigue score improved significantly between T1 and T2 (median change –2 points, IQR –7 to 3; *P*<.001) (Table 2), whereas no significant differences were found in the proportions of normal, mild, or severe fatigue (*P*=.12). An overview of the proportions of patients with normal, mild, and severe fatigue at T1 and T2, the proportional flow, and the direction of change can be found in Figure 1. In addition, Multimedia Appendix 6 shows the proportion, flow, and direction of the change of fatigue stratified for the type of diagnosis (ie, hospitalized and nonhospitalized test-diagnosed patients, physician-diagnosed patients, and patients without a formal diagnosis/test).

### Self-constructed Physical and Mental Fatigue Questions

**Figure 2:**
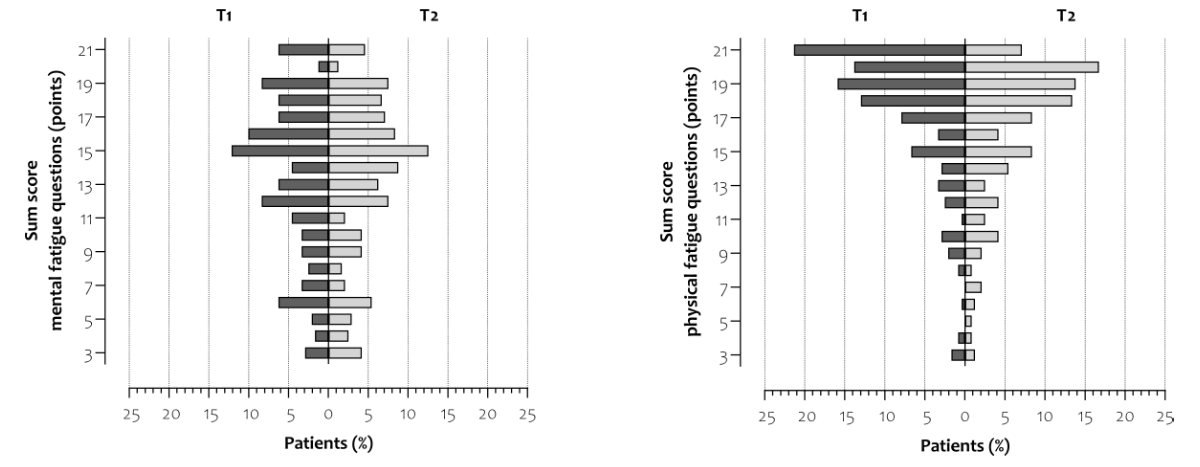
The distribution of patients with long COVID who have confirmed COVID-19 across the spectrum of self-constructed mental (left) and physical (right) fatigue at on average 10 (T1) and 23 (T2) weeks after onset of symptoms (n=239). Scores range from 3 to 21 points, and higher scores indicate higher levels of fatigue. T1: time of completing the first survey; T2: time of completing the second survey.

Patients with confirmed COVID-19 reported median physical and mental fatigue scores of 19 points (IQR 16-20) and 15 points (IQR 10-17) at T1. Between T1 and T2, a significant decrease was found in physical fatigue score (median change –1 point, IQR –3 to 0; *P*<.001), but not in mental fatigue score (median change 0 points, IQR –3 to 3; *P*=.52) (Table 2). Figure 2 shows the distributions of patients across the spectrum of physical and mental fatigue at T1 and T2.

## Discussion

### Principal Findings

To the best of our knowledge, this is the first study to measure fatigue over time in members from online long COVID peer support groups with confirmed COVID-19 using a validated and standardized measurement with generic cutoff values to determine normal, mild, and severe fatigue. Our study indicates that severe fatigue is highly prevalent in patients with long COVID at approximately 3 and 6 months after the infection. Furthermore, our longitudinal follow-up data suggest that fatigue does not resolve over time in all patients, even if they receive health care. In addition, patients experience both physical and mental fatigue.

Fatigue is the most prominent symptom in patients with long COVID [2,4], irrespective of the severity of the initial infection [14]. Nevertheless, most studies are cross-sectional and use a binary question (eg, fatigued/not fatigued) to assess the prevalence of fatigue [2,16,39]. Therefore, little is known about the change in fatigue intensity over time [14,40]. Our study used a validated and standardized questionnaire to assess fatigue and was able to quantify fatigue intensity. Indeed, fatigue is highly prevalent in our sample. Moreover, fatigue was reported to be generally high. The median fatigue scores found in our sample are equal to or higher than those of other chronic diseases that are characterized by fatigue, such as chronic obstructive pulmonary disease [41], asthma [42], Q fever [20], multiple sclerosis [43], rheumatoid arthritis [44], or systemic sclerosis [45]. These findings are remarkable for such a young population with few self-reported comorbidities and good self-reported health status before the infection. Previously, other viral and nonviral infections have been linked to prolonged and debilitating fatigue [20,46-50]. For example, Lam and colleagues investigated long-term complaints in SARS survivors and found that approximately one-third of SARS survivors met the modified 1994 US Centers for Disease Control and Prevention criteria for chronic fatigue syndrome more than 3 years after having SARS [17]. Moreover, MERS survivors often experience chronic fatigue [19]. For patients with long COVID, it remains unclear whether fatigue will resolve spontaneously in the longer term. Our follow-up data show little to no improvement in the proportion of patients with severe fatigue between 3 and 6 months, despite receiving medical and allied health care. Consequently, almost two-thirds of the patients in our sample are progressing toward chronic fatigue (ie, severe fatigue that persists longer than six months [51]). The fact that some patients may experience debilitating chronic fatigue is worrisome and could have a major long-term impact upon these individuals as well as on the health care system and society as a whole [10,11,52]. Indeed, fatigue is strongly related to health-related QoL and aspects of day-to-day life [14,25,53,54], and it often involves sick leave, increased health care consumption, and more hidden costs, such as informal care by friends or family members [55-57].

Fatigue is a complex and challenging symptom, as multiple factors can play a role in the initiation and maintenance of fatigue, as seen in other chronic diseases [58]. It can present itself as mental fatigue, physical fatigue, or both [40]. Therefore, a patient-tailored treatment based upon a holistic and comprehensive assessment of systemic, physical, psychological, and behavioral factors is proposed to alleviate the fatigue symptom burden [59]. To date, it remains unknown which treatment strategies are effective to improve fatigue in patients with long COVID. Several treatment strategies for fatigue are proposed based upon knowledge from the fast-growing evidence regarding COVID-19 and other pathologies, such as multidisciplinary rehabilitation, energy conservation techniques, pacing, cognitive behavioral therapy, graded exercise therapy, or physical training [25,54,60-65]. Future research needs to provide evidence regarding underlying pathways, evaluate the effectiveness of existing treatment strategies, and identify susceptible candidates, as it is expected that not everyone will benefit from the same treatment strategy due to the multifactorial nature of fatigue. Moreover, anecdotal evidence shows that patients report having within-day and between-day variations in their daily symptoms, including fatigue [54,66,67]; these cannot be captured in detail by completing a questionnaire once or twice over a longer period of time. In this, the use of an ecological momentary assessment may be valuable, as this approach involves repeated measurements of the participant’s symptoms, behavior, and context in vivo and in real time [68]. More insights in diurnal variation in fatigue and its association with other symptoms may be useful in the development of more tailored treatment strategies for fatigue in patients with long COVID.

### Methodological Considerations and Limitations

The current study has several limitations. First, the survey was only made available to members of online long COVID peer support groups. This probably caused selection bias, as it is reasonable to assume that patients with high symptom burden are more likely to become members of online long COVID peer support groups. Second, all results were collected using a web-based survey. Therefore, besides the self-reported symptoms, the patients’ height, body weight, and medical status before and during the infection were also based on self-report, which may have affected the internal validity of the current findings to some extent. Recently, the National Institute for Health and Care Excellence [69] proposed a case definition of long COVID whereby alternative diagnosis should be excluded when identifying patients with long COVID. Due to the nature and timing of this study (ie, early phase of the pandemic), this was not possible in the current study. Third, approximately 1 out of 3 participants who consented to be approached for follow-up research did not respond to the second wave of the survey. The authors have no information about the possible reasons for not responding to the second wave of the survey, although a between-group comparison was made to find possible differences [11]. Fourth, the majority of our sample were women, which limits our external validity. Nevertheless, evidence is growing that women are more prone to develop long COVID [70]. Fifth, self-constructed questions were used to quantify mental fatigue, although validated questionnaires (such as the Chalder fatigue index) to assess mental (and physical) fatigue are available [71]. Therefore, no definite conclusions on the burden of mental fatigue in online long COVID peer support groups can be drawn based on the current study. Nevertheless, the current results indicate that COVID-19 can impact both physical and mental fatigue in the long term. Furthermore, this study was conducted in adults, although evidence regarding long COVID in children and adolescents is starting to emerge [72,73].

### Conclusions

Severe fatigue is highly prevalent in members of online long COVID peer support groups both at approximately 3 and 6 months after onset of symptoms. As not enough time has passed since the start of the COVID-19 pandemic, it is unclear whether this fatigue will resolve spontaneously in the longer term. Future research needs to focus on the prognosis, possible causes, and treatment strategies for physical and mental fatigue in patients with long COVID.

## Appendix

Multimedia Appendix 1 Members of online long COVID peer support groups with presumed COVID-19 (n=766): characteristics, received health care, and fatigue-related measures.

Multimedia Appendix 2 Additional information regarding self-reported pre-existing comorbidities and symptoms during the acute phase of COVID-19 and at the moment of completing the surveys.

Multimedia Appendix 3 The Checklist Individual Strength–subscale subjective fatigue.

Multimedia Appendix 4 Self-constructed physical and mental fatigue based upon 3 items of the Checklist Individual Strength–subscale subjective fatigue.

Multimedia Appendix 5 Flowchart of participants’ inclusion.

Multimedia Appendix 6 Prevalence of normal, mild, and severe fatigue using the Checklist Individual Strength–subscale subjective fatigue at T1 and T2, the proportional flow, and the direction of change of fatigue stratified for type of diagnosis.

## Abbreviations

CIS-Fatigue: Checklist Individual Strength–subscale subjective fatigue
CT: computed tomography
GP: general practitioner
ICU: intensive care unit
MERS: Middle East respiratory syndrome
OT: occupational therapist
PCR: polymerase chain reaction
PT: physiotherapist
QoL: quality of life
SARS: severe acute respiratory syndrome
STROBE: Strengthening the Reporting of Observational Studies in Epidemiology
T1: time point of completing the first survey
T2: time point of completing the second survey
