# Supplementary material for: Severe Fatigue in Long COVID: Web-Based Quantitative Follow-up Study in Members of Online Long COVID Support Groups
Source: J Med Internet Res. 2021 Sep 21;23(9):e30274. doi: 10.2196/30274 (PMC8457337; doi:10.2196/30274)
Supplement: Multimedia Appendix 1 [file jmir_v23i9e30274_app1.docx]

**Multimedia Appendix 1**

**Severe Fatigue in Long COVID: Web-Based Quantitative Follow-up Study in Members of Online Long COVID Support Groups**

Maarten Van Herck^1,2,3,4*^, Yvonne M.J. Goërtz^2,3,4*^, Sarah Houben-Wilke^2^, Felipe V.C. Machado^2,3,4^, Roy Meys^2,3,4^, Jeannet M. Delbressine^2^, Anouk W. Vaes^2^, Chris Burtin^1^, Rein Posthuma^2,3,4^, Frits M.E. Franssen^2,3,4^, Bita Hajian^2^, Herman Vijlbrief^5^, Yvonne Spies^5^, Alex J. van ’t Hul^6^, Daisy J.A. Janssen^2,7^, Martijn A. Spruit^2,3,4^

* shared first author

**Affiliations**

^1^ REVAL – Rehabilitation Research Center, BIOMED – Biomedical Research Institute, Faculty of Rehabilitation Sciences, Hasselt University, Diepenbeek, Belgium

^2^ Department of Research and Development, Ciro, Horn, the Netherlands

^3^ Nutrim School of Nutrition and Translational Research in Metabolism, Faculty of Health, Medicine and Life Sciences, Maastricht University, Maastricht, the Netherlands

^4^ Department of Respiratory Medicine, Maastricht University Medical Centre (MUMC+), Maastricht, the Netherlands

^5^ Lung Foundation Netherlands, Amersfoort, the Netherlands

^6^ Department of Pulmonary Disease, Radboud University Medical Center, Nijmegen, the Netherlands

^7^ Department of Health Services Research, Care and Public Health Research Institute, Faculty of Health, Medicine and Life Sciences, Maastricht University, Maastricht, the Netherlands

## Multimedia Appendix 1

## Members from online long COVID peer support groups with presumed COVID-19 (*n*=766): characteristics, received healthcare, and fatigue-related measures

In general, the results of the 766 members from online long COVID peer support groups with presumed COVID-19 were very similar to those with test-diagnosed COVID-19 (e.g. mostly middle-aged women, majority had no comorbidities and a good self-reported health status before infection, etc.; Table 1 Multimedia Appendix 1).

Table 1 Multimedia Appendix 1. Characteristics of patients with presumed COVID-19

|  | **Presumed COVID-19 patients (*n*=766)** |
| --- | --- |
| **Women, *n* (%)** | 652 (85.1) |
| **Age, years (*m*, [IQR])** | 48.0 [40.0-54.0] |
| **BMI, kg/m^2^ (*m*, [IQR])** | 24.7 [22.2-28.1] |
| **Time between symptoms onset and T1 survey, weeks (mean ±SD)** | 11.5 ±2.1 |
| **Time between symptoms onset and T2 survey, weeks (mean ±SD)** | 23.8 ±2.1 |
| **Married/living with partner, *n* (%)** | 543 (70.9) |
| **Educational level, *n* (%)** |  |
| Low | 19 (2.5) |
| Medium | 258 (33.7) |
| High | 484 (63.2) |
| Other/don’t want to say | 5 (0.7) |
| **Pre-existing comorbidities, *n* (%)** |  |
| None | 475 (62.0) |
| 1 comorbidity | 196 (25.6) |
| ≥2 comorbidities | 95 (12.4) |
| **Health status before infection, *n* (%)** |  |
| Good | 653 (85.2) |
| Moderate | 107 (14.0) |
| Poor | 6 (0.8) |
| **Health status at T1, *n* (%)** | |
| Good | 33 (4.3) |
| Moderate | 469 (61.2) |
| Poor | 264 (34.5) |
| **Number of symptoms (*m*, [IQR])** |  |
| During acute infection | 14 [11-18] |
| At T1 | 7 [4-9] |
| At T2 | 6 [4-9] |
| *Abbreviations: BMI, body mass index; IQR, Interquartile range, n, number; m, median; SD, standard deviation; T1, timepoint of completing the 1^st^ survey; T2, timepoint of completing the 2^nd^ survey.* | |

At T1 a median CIS-Fatigue score of 50 [44-53] points was reported with 4% and 93% of the presumed COVID-19 patients having mild and severe fatigue, respectively. Median self-constructed mental and physical fatigue were respectively 15 [9-18] points and 19 [17-21] points at T1. Significant improvements were found in median CIS-Fatigue score (median change: -2 [-7 to 1] points, *P*<.001) and self-constructed mental (median change: 0 [-4 to 2] points, *P*=.001) and physical fatigue (median change: -1 [-3 to 0] points, *P*<.001). Furthermore, changes in the prevalence of fatigue were not symmetrical between T1 and T2 (χ2(3, N = 766) = 63.7, *P*<.001). A post-hoc analysis showed that the changes from severe to normal and normal to severe fatigue, and the change from severe to mild and mild to severe fatigue were not symmetric (both Bonferroni-adjusted *P*-values <.001). An overview of the fatigue-related measures and received care in members from online long COVID peer support groups with presumed COVID-19 at T1 and T2 can be found in Table 2 Multimedia Appendix 1.

Table 2 Multimedia Appendix 1. Fatigue-related measures and received healthcare in patients with presumed COVID-19

|  |  | **Presumed COVID-19 patients (*n*=766)** | | |
| --- | --- | --- | --- | --- |
|  |  | **T1** | **T2** | ***P*-value** |
| **Fatigue-related measures** | | | |  |
| General fatigue, CIS-Fatigue, points (*m*, [IQR]) | | 50 [44-53] | 47 [40-52] | <.001 |
| Severe fatigue, *n* (%) | | 715 (93.3) | 643 (83.9) | <.001 |
| Mental fatigue questions, points (*m*, [IQR]) | | 15 [9-18] | 14 [8-17] | .001 |
| Physical fatigue questions, points (*m*, [IQR]) | | 19 [17-21] | 18 [15-20] | <.001 |
| **Received health care – Medical profession, *n* (%)** | | | |  |
| Received care from ≥1 medical profession | | 522 (68.1) | 661 (86.3) | <.001 |
| General practitioner | | 503 (65.7) | 629 (82.1) | <.001 |
| Medical specialist | | 137 (17.9) | 372 (48.6) | <.001 |
| Nurse | | 12 (1.6) | 31 (4.0) | <.001 |
| **Received health care – AHP, *n* (%)** | | | |  |
| Received care from ≥1 AHP | | 214 (27.9) | 487 (63.6) | <.001 |
| Physiotherapist | | 186 (24.3) | 459 (59.9) | <.001 |
| Psychologist | | 44 (5.7) | 102 (13.3) | <.001 |
| Occupational therapist | | 14 (1.8) | 70 (9.1) | <.001 |
| Dietician | | 30 (3.9) | 102 (13.3) | <.001 |
| Speech and language therapist | | 3 (0.4) | 46 (6.0) | <.001 |
| **Rehabilitation (in- or outpatient), *n* (%)** | | 10 (1.3) | 40 (5.2) | <.001 |
| *Abbreviations:* *AHP, Allied Health care Professional;* *CIS-Fatigue, Checklist Individual Strength - subscale subjective fatigue; IQR, Interquartile range; m, median; n, number; T1, at time of completing the 1^st^ survey; T2, at time of completing the 2^nd^ survey.* | | | | |
