# Supplementary material for: Severe Fatigue in Long COVID: Web-Based Quantitative Follow-up Study in Members of Online Long COVID Support Groups
Source: J Med Internet Res. 2021 Sep 21;23(9):e30274. doi: 10.2196/30274 (PMC8457337; doi:10.2196/30274)
Supplement: Multimedia Appendix 2 [file jmir_v23i9e30274_app2.docx]

**Multimedia Appendix 2**

**Severe Fatigue in Long COVID: Web-Based Quantitative Follow-up Study in Members of Online Long COVID Support Groups**

Maarten Van Herck^1,2,3,4*^, Yvonne M.J. Goërtz^2,3,4*^, Sarah Houben-Wilke^2^, Felipe V.C. Machado^2,3,4^, Roy Meys^2,3,4^, Jeannet M. Delbressine^2^, Anouk W. Vaes^2^, Chris Burtin^1^, Rein Posthuma^2,3,4^, Frits M.E. Franssen^2,3,4^, Bita Hajian^2^, Herman Vijlbrief^5^, Yvonne Spies^5^, Alex J. van ’t Hul^6^, Daisy J.A. Janssen^2,7^, Martijn A. Spruit^2,3,4^

* shared first author

**Affiliations**

^1^ REVAL – Rehabilitation Research Center, BIOMED – Biomedical Research Institute, Faculty of Rehabilitation Sciences, Hasselt University, Diepenbeek, Belgium

^2^ Department of Research and Development, Ciro, Horn, the Netherlands

^3^ Nutrim School of Nutrition and Translational Research in Metabolism, Faculty of Health, Medicine and Life Sciences, Maastricht University, Maastricht, the Netherlands

^4^ Department of Respiratory Medicine, Maastricht University Medical Centre (MUMC+), Maastricht, the Netherlands

^5^ Lung Foundation Netherlands, Amersfoort, the Netherlands

^6^ Department of Pulmonary Disease, Radboud University Medical Center, Nijmegen, the Netherlands

^7^ Department of Health Services Research, Care and Public Health Research Institute, Faculty of Health, Medicine and Life Sciences, Maastricht University, Maastricht, the Netherlands

## Multimedia Appendix 2

## Extra information regarding self-reported pre-existing comorbidities and symptoms during acute phase of COVID-19 and at the moment of completing the surveys

**Self-reported pre-existing comorbidities**

Participants were asked to self-report the (chronic) diseases they were diagnosed with before COVID-19 from a pre-defined list of 15 (chronic) medical conditions. The 15 chronic diseases were carefully selected from a broader list of chronic diseases by the authors of the current manuscript and included: diabetes mellitus; lung disease; cancer; heart disease; renal disease; anxiety disorder; mood disorder; hypertension; obesity; rheumatic disease; Parkinson disease; stroke; osteoporosis; migraine; and disorder related to emotional exhaustion. Moreover, there was the option of an open text field to add other disorders.

**Symptoms during acute phase of COVID-19 and at the moment of completing the surveys**

In total, 29 symptoms were assessed using self-report. The symptom list was carefully selected by scientists, methodologists, healthcare professionals and COVID-19 patients from the online peer support groups and consisted of the symptoms: body temperature (37.0–37.9°C); fever (body temperature ⩾38.0°C); cough; mucus; nose cold; sneezing; dyspnoea; sore throat; fatigue; muscle pain; joint pain; anosmia; ageusia; headache; dizziness; diarrhoea; nausea; vomiting; red spots on toes/feet; pain/burning feeling in the lungs; ear pain; chest tightness; pain between shoulder blades; heart palpitations; increased resting heart rate; eye problems; sudden loss of body weight; burning feeling in the trachea; and heat flushes. Moreover, there was the option of an open text field to add other symptoms.
