# Supplementary material for: Severe Fatigue in Long COVID: Web-Based Quantitative Follow-up Study in Members of Online Long COVID Support Groups
Source: J Med Internet Res. 2021 Sep 21;23(9):e30274. doi: 10.2196/30274 (PMC8457337; doi:10.2196/30274)
Supplement: Multimedia Appendix 3 [file jmir_v23i9e30274_app3.docx]

**Multimedia Appendix 3**

**Severe Fatigue in Long COVID: Web-Based Quantitative Follow-up Study in Members of Online Long COVID Support Groups**

Maarten Van Herck^1,2,3,4*^, Yvonne M.J. Goërtz^2,3,4*^, Sarah Houben-Wilke^2^, Felipe V.C. Machado^2,3,4^, Roy Meys^2,3,4^, Jeannet M. Delbressine^2^, Anouk W. Vaes^2^, Chris Burtin^1^, Rein Posthuma^2,3,4^, Frits M.E. Franssen^2,3,4^, Bita Hajian^2^, Herman Vijlbrief^5^, Yvonne Spies^5^, Alex J. van ’t Hul^6^, Daisy J.A. Janssen^2,7^, Martijn A. Spruit^2,3,4^

* shared first author

**Affiliations**

^1^ REVAL – Rehabilitation Research Center, BIOMED – Biomedical Research Institute, Faculty of Rehabilitation Sciences, Hasselt University, Diepenbeek, Belgium

^2^ Department of Research and Development, Ciro, Horn, the Netherlands

^3^ Nutrim School of Nutrition and Translational Research in Metabolism, Faculty of Health, Medicine and Life Sciences, Maastricht University, Maastricht, the Netherlands

^4^ Department of Respiratory Medicine, Maastricht University Medical Centre (MUMC+), Maastricht, the Netherlands

^5^ Lung Foundation Netherlands, Amersfoort, the Netherlands

^6^ Department of Pulmonary Disease, Radboud University Medical Center, Nijmegen, the Netherlands

^7^ Department of Health Services Research, Care and Public Health Research Institute, Faculty of Health, Medicine and Life Sciences, Maastricht University, Maastricht, the Netherlands

## Multimedia Appendix 3

## The Checklist Individual Strength – Subscale subjective fatigue (CIS-Fatigue)

********* CIS_08 *********

**Checklist Individual Strength**

**Radboud University Nijmegen Medical Centre, The Netherlands**

Instruction:

On the next page you find 8 statements. With these statements we wish to get an impression of how you have felt during the last two weeks. For example:

**I feel relaxed**

| **yes,** that is true | **X** |  |  |  |  |  |  | **no,** that is not true |
| --- | --- | --- | --- | --- | --- | --- | --- | --- |

If you feel that this statement is entirely true, tick the left box; as follows:

**I feel relaxed**

| **yes,** that is true |  |  |  |  |  |  | **X** | **no,** that is not true |
| --- | --- | --- | --- | --- | --- | --- | --- | --- |

If you feel that this statement is not true at all, tick the right box; as follows:

**I feel relaxed**

If you feel that this statement is neither "yes, that is true", nor "no, that is not true", tick the box that is most in accordance with how you have felt.

For example, if you feel relaxed, but not very relaxed, tick one of the boxes close to "yes, that is true": as follows:

| **yes,** that is true |  |  | **X** |  |  |  |  | **no,** that is not true |
| --- | --- | --- | --- | --- | --- | --- | --- | --- |

**I feel relaxed**

Do not skip any statement and tick each statement only once.

| 1. | I feel tired. | **yes, that is true** |  |  |  |  |  |  |  | **no, that is not true** |
| --- | --- | --- | --- | --- | --- | --- | --- | --- | --- | --- |

| 2. | Physically I feel exhausted. | **yes, that is true** |  |  |  |  |  |  |  | **no, that is not true** |
| --- | --- | --- | --- | --- | --- | --- | --- | --- | --- | --- |

| 3. | I feel fit. | **yes, that is true** |  |  |  |  |  |  |  | **no, that is not true** |
| --- | --- | --- | --- | --- | --- | --- | --- | --- | --- | --- |

| 4. | I feel powerless. | **yes, that is true** |  |  |  |  |  |  |  | **no, that is not true** |
| --- | --- | --- | --- | --- | --- | --- | --- | --- | --- | --- |

| 5. | I am rested. | **yes, that is true** |  |  |  |  |  |  |  | **no, that is not true** |
| --- | --- | --- | --- | --- | --- | --- | --- | --- | --- | --- |

| 6. | Physically I feel I am in bad form. | **yes, that is true** |  |  |  |  |  |  |  | **no, that is not true** |
| --- | --- | --- | --- | --- | --- | --- | --- | --- | --- | --- |

| 7. | I tire easily. | **yes, that is true** |  |  |  |  |  |  |  | **no, that is not true** |
| --- | --- | --- | --- | --- | --- | --- | --- | --- | --- | --- |

| 8. | Physically I feel I am in an excellent condition. | **yes, that is true** |  |  |  |  |  |  |  | **no, that is not true** |
| --- | --- | --- | --- | --- | --- | --- | --- | --- | --- | --- |
