# Supplementary material for: Severe Fatigue in Long COVID: Web-Based Quantitative Follow-up Study in Members of Online Long COVID Support Groups
Source: J Med Internet Res. 2021 Sep 21;23(9):e30274. doi: 10.2196/30274 (PMC8457337; doi:10.2196/30274)
Supplement: Multimedia Appendix 4 [file jmir_v23i9e30274_app4.docx]

**Multimedia Appendix 4**

**Severe Fatigue in Long COVID: Web-Based Quantitative Follow-up Study in Members of Online Long COVID Support Groups**

Maarten Van Herck^1,2,3,4*^, Yvonne M.J. Goërtz^2,3,4*^, Sarah Houben-Wilke^2^, Felipe V.C. Machado^2,3,4^, Roy Meys^2,3,4^, Jeannet M. Delbressine^2^, Anouk W. Vaes^2^, Chris Burtin^1^, Rein Posthuma^2,3,4^, Frits M.E. Franssen^2,3,4^, Bita Hajian^2^, Herman Vijlbrief^5^, Yvonne Spies^5^, Alex J. van ’t Hul^6^, Daisy J.A. Janssen^2,7^, Martijn A. Spruit^2,3,4^

* shared first author

**Affiliations**

^1^ REVAL – Rehabilitation Research Center, BIOMED – Biomedical Research Institute, Faculty of Rehabilitation Sciences, Hasselt University, Diepenbeek, Belgium

^2^ Department of Research and Development, Ciro, Horn, the Netherlands

^3^ Nutrim School of Nutrition and Translational Research in Metabolism, Faculty of Health, Medicine and Life Sciences, Maastricht University, Maastricht, the Netherlands

^4^ Department of Respiratory Medicine, Maastricht University Medical Centre (MUMC+), Maastricht, the Netherlands

^5^ Lung Foundation Netherlands, Amersfoort, the Netherlands

^6^ Department of Pulmonary Disease, Radboud University Medical Center, Nijmegen, the Netherlands

^7^ Department of Health Services Research, Care and Public Health Research Institute, Faculty of Health, Medicine and Life Sciences, Maastricht University, Maastricht, the Netherlands

## Multimedia Appendix 4

## Self-constructed physical and mental fatigue based upon three items of the CIS-Fatigue

**Physical fatigue**

| 1. | Physically I feel exhausted. | **yes, that is true** |  |  |  |  |  |  |  | **no, that is not true** |
| --- | --- | --- | --- | --- | --- | --- | --- | --- | --- | --- |

| 2. | Physically I feel I am in bad form. | **yes, that is true** |  |  |  |  |  |  |  | **no, that is not true** |
| --- | --- | --- | --- | --- | --- | --- | --- | --- | --- | --- |

| 3. | Physically I feel I am in an excellent condition. | **yes, that is true** |  |  |  |  |  |  |  | **no, that is not true** |
| --- | --- | --- | --- | --- | --- | --- | --- | --- | --- | --- |

**Mental fatigue**

| 1. | Mentally I feel exhausted. | **yes, that is true** |  |  |  |  |  |  |  | **no, that is not true** |
| --- | --- | --- | --- | --- | --- | --- | --- | --- | --- | --- |

| 2. | Mentally I feel I am in bad form. | **yes, that is true** |  |  |  |  |  |  |  | **no, that is not true** |
| --- | --- | --- | --- | --- | --- | --- | --- | --- | --- | --- |

| 3. | Mentally I feel I am in an excellent condition. | **yes, that is true** |  |  |  |  |  |  |  | **no, that is not true** |
| --- | --- | --- | --- | --- | --- | --- | --- | --- | --- | --- |

**Additional clarification regarding the self-constructed physical and mental fatigue questions**

Each question is scored on a seven-point Likert scale ranging from 1 (“Yes, that is true”) to 7 points (“No, that is not true”). The total mental and physical fatigue score can be calculated by summing up all self-constructed mental / physical fatigue questions. Though, the negatively framed questions ‘Physically/Mentally I Feel exhausted’ and ‘Physically/Mentally I feel I am in a bad condition’ should be recoded as higher scores indicate worse/more physical and mental fatigue. The physical and mental fatigue scores range from 3 to 21 points, and a higher score indicates worse physical and mental fatigue, respectively.
