# Supplementary material for: Severe Fatigue in Long COVID: Web-Based Quantitative Follow-up Study in Members of Online Long COVID Support Groups
Source: J Med Internet Res. 2021 Sep 21;23(9):e30274. doi: 10.2196/30274 (PMC8457337; doi:10.2196/30274)

**Multimedia Appendix 5**

**Severe Fatigue in Long COVID: Web-Based Quantitative Follow-up Study in Members of Online Long COVID Support Groups**

Maarten Van Herck^1,2,3,4*^, Yvonne M.J. Goërtz^2,3,4*^, Sarah Houben-Wilke^2^, Felipe V.C. Machado^2,3,4^, Roy Meys^2,3,4^, Jeannet M. Delbressine^2^, Anouk W. Vaes^2^, Chris Burtin^1^, Rein Posthuma^2,3,4^, Frits M.E. Franssen^2,3,4^, Bita Hajian^2^, Herman Vijlbrief^5^, Yvonne Spies^5^, Alex J. van ’t Hul^6^, Daisy J.A. Janssen^2,7^, Martijn A. Spruit^2,3,4^

* shared first author

**Affiliations**

^1^ REVAL – Rehabilitation Research Center, BIOMED – Biomedical Research Institute, Faculty of Rehabilitation Sciences, Hasselt University, Diepenbeek, Belgium

^2^ Department of Research and Development, Ciro, Horn, the Netherlands

^3^ Nutrim School of Nutrition and Translational Research in Metabolism, Faculty of Health, Medicine and Life Sciences, Maastricht University, Maastricht, the Netherlands

^4^ Department of Respiratory Medicine, Maastricht University Medical Centre (MUMC+), Maastricht, the Netherlands

^5^ Lung Foundation Netherlands, Amersfoort, the Netherlands

^6^ Department of Pulmonary Disease, Radboud University Medical Center, Nijmegen, the Netherlands

^7^ Department of Health Services Research, Care and Public Health Research Institute, Faculty of Health, Medicine and Life Sciences, Maastricht University, Maastricht, the Netherlands

**Figure 1 Multimedia Appendix 5**. Flowchart of participants’ inclusion. Abbreviations: COVID-19, coronavirus disease 2019; ICU, Intensive Care Unit; n, number of subjects.


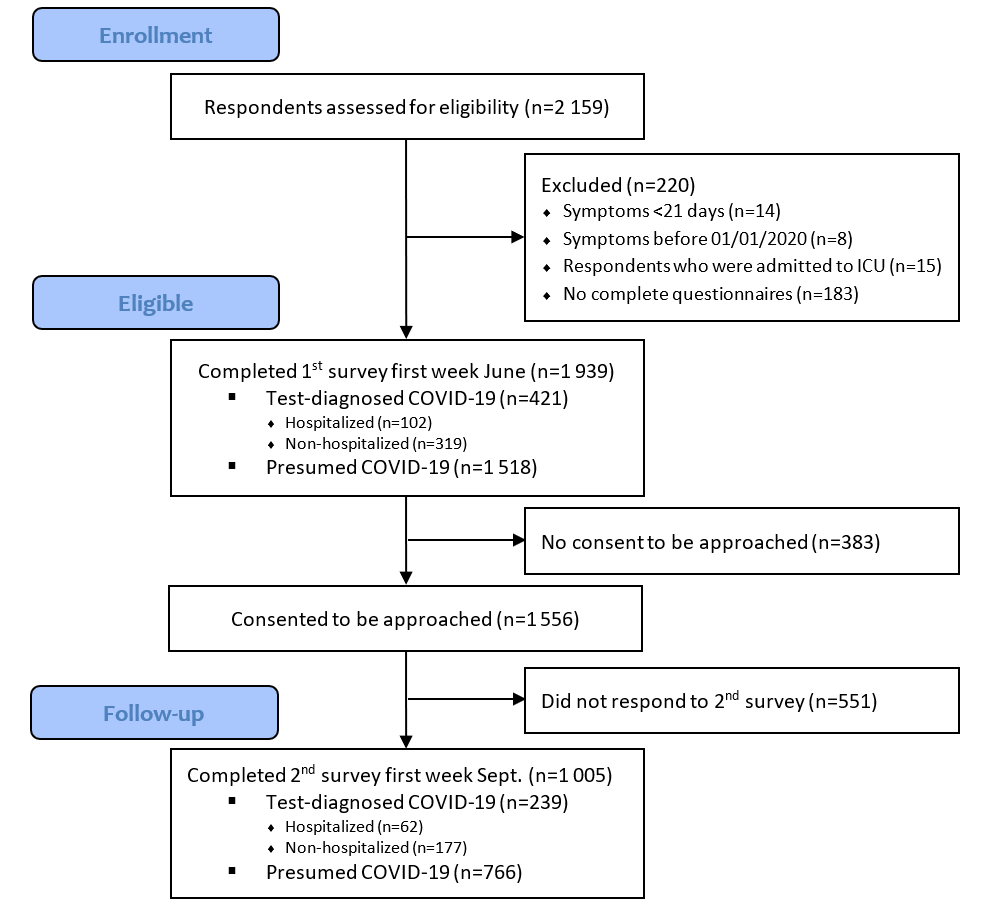

Supplement: Multimedia Appendix 5 [file jmir_v23i9e30274_app5.docx]
