# Supplementary material for: Severe Fatigue in Long COVID: Web-Based Quantitative Follow-up Study in Members of Online Long COVID Support Groups
Source: J Med Internet Res. 2021 Sep 21;23(9):e30274. doi: 10.2196/30274 (PMC8457337; doi:10.2196/30274)
Supplement: Multimedia Appendix 6 [file jmir_v23i9e30274_app6.docx]

**Multimedia Appendix 6**

**Severe Fatigue in Long COVID: Web-Based Quantitative Follow-up Study in Members of Online Long COVID Support Groups**

Maarten Van Herck^1,2,3,4*^, Yvonne M.J. Goërtz^2,3,4*^, Sarah Houben-Wilke^2^, Felipe V.C. Machado^2,3,4^, Roy Meys^2,3,4^, Jeannet M. Delbressine^2^, Anouk W. Vaes^2^, Chris Burtin^1^, Rein Posthuma^2,3,4^, Frits M.E. Franssen^2,3,4^, Bita Hajian^2^, Herman Vijlbrief^5^, Yvonne Spies^5^, Alex J. van ’t Hul^6^, Daisy J.A. Janssen^2,7^, Martijn A. Spruit^2,3,4^

* shared first author

**Affiliations**

^1^ REVAL – Rehabilitation Research Center, BIOMED – Biomedical Research Institute, Faculty of Rehabilitation Sciences, Hasselt University, Diepenbeek, Belgium

^2^ Department of Research and Development, Ciro, Horn, the Netherlands

^3^ Nutrim School of Nutrition and Translational Research in Metabolism, Faculty of Health, Medicine and Life Sciences, Maastricht University, Maastricht, the Netherlands

^4^ Department of Respiratory Medicine, Maastricht University Medical Centre (MUMC+), Maastricht, the Netherlands

^5^ Lung Foundation Netherlands, Amersfoort, the Netherlands

^6^ Department of Pulmonary Disease, Radboud University Medical Center, Nijmegen, the Netherlands

^7^ Department of Health Services Research, Care and Public Health Research Institute, Faculty of Health, Medicine and Life Sciences, Maastricht University, Maastricht, the Netherlands

**Figure 1 Multimedia Appendix 6.** Prevalence of normal, mild, and severe fatigue using CIS-Fatigue at T1 and T2 (approximately 3 and 6 months after onset of symptoms, respectively), the proportional flow, and direction of change of fatigue stratified for type of diagnosis: (A) hospitalized test-diagnosed (n=62), (B) non-hospitalized test-diagnosed (n=177), (C) doctor-diagnosed (n=454), and (D) no formal diagnosis/testing (n=312).


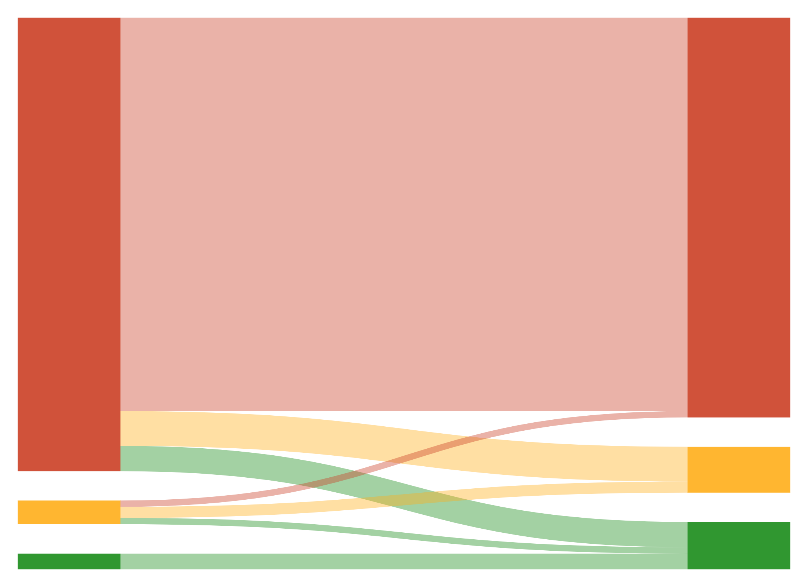

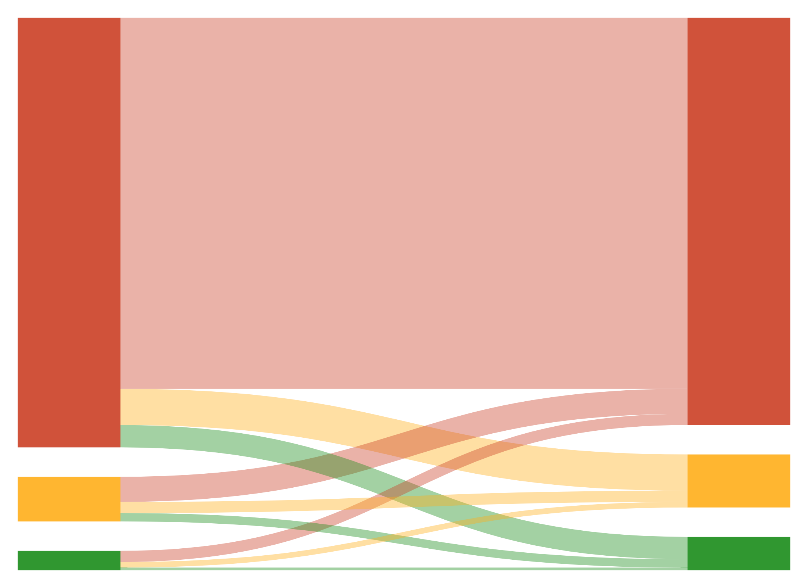

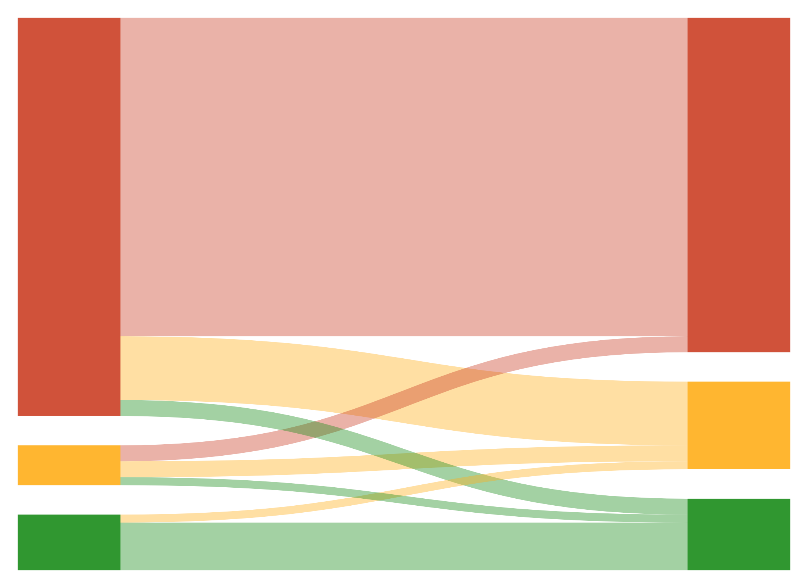


**B**

**D**


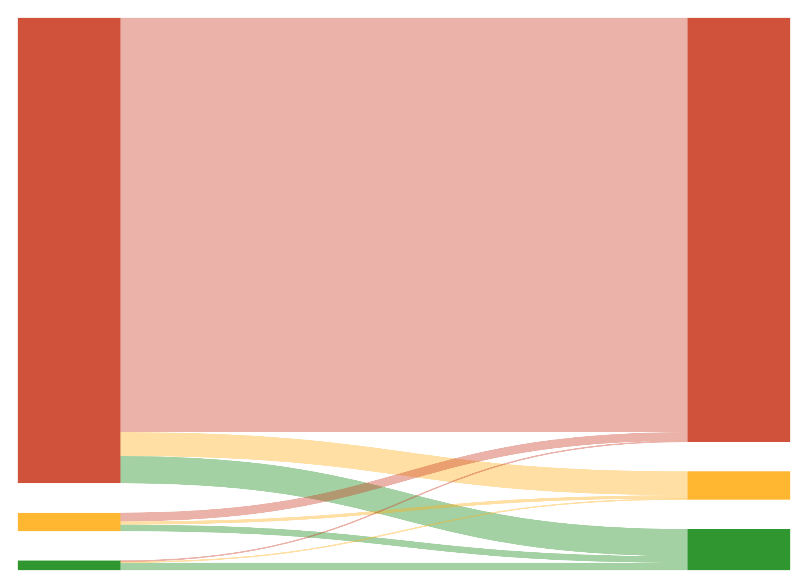


**5.7%** (26)

**8.4%** (38)

**9.0%** (16)

**6.8%** (12)

**9.3%** (29)

**9.6%** (30)

**3.2%** (10)

**4.8%** (15)

**67.7%**

(42)

**85.9%**

(390)

**3.7%** (17)

**87.0%**

(154)

**81.1%**

(253)

%

**92.0%**

(287)

%

**94.3%**

(428)

**82.5%**

(146)

**80.6%**

(50)

**A**

**C**

Mild fatigue

Severe fatigue

Normal fatigue

**4.0%** (7)

**11.3%** (7)

**2.0%** (9)

**10.7%** (19)

**T2**

**T1**

**14.5%**

(9)

**T2**

**T2**

**T2**

**T1**

**T1**

**T1**

**17.7%**

(11)

**8.1%** (5)

Additional clarification regarding the Sankey diagrams presented above: Relative numbers (in bold) and absolute numbers (in brackets) are presented in each graph. The width of the lines is proportional to the flow rate. In other words: the wider the line, the more participants/higher proportion followed that trajectory. Abbreviations: CIS-Fatigue, Checklist Individual Strength - subscale subjective fatigue; T1, time of completing the 1^st^ survey; T2, time of completing the 2^nd^ survey.
